# Genetic, age, and diet effects on phytate degradation of laying hens studied in combined *in vivo* and *in vitro* assays

**DOI:** 10.3389/fphys.2026.1730157

**Published:** 2026-03-09

**Authors:** Anna Hanauska, Vera Sommerfeld, Markus Schmid, Valentin P. Haas, Korinna Huber, Jörn Bennewitz, Markus Rodehutscord

**Affiliations:** Institute of Animal Science, University of Hohenheim, Stuttgart, Germany

**Keywords:** age, genetic strain, heritability, *in vitro* assay, laying hen, mucosa, phosphorus, phytate degradation

## Abstract

Endogenous mucosal phosphatases of chicken degrade phytate from the feed to a variable extent. The objective of this study was to investigate endogenous mucosal phosphatases as affected by laying hen strain, hen age, and dietary phosphorus (P) renunciation. Two cohorts of the strains Lohmann Brown-classic (LB) and Lohmann LSL-classic (LSL) were fed for 3 weeks, starting in week 27 and 39 of age, respectively, one of two diets with (P+) or without (P−) mineral P supplement. Total excreta were collected. In weeks 30 and 42, hens were sacrificed, and ileal digesta and duodenum mucosa were collected. Mucosa was freeze-dried before being used in a three-step *in vitro* assay. The concentrations of InsP isomers, *myo*-inositol (MI), P, and calcium (Ca) were measured in the excreta and digesta. InsP isomers were measured in the *in vitro* incubation residues. Most of the excreta InsP concentrations were lower in LB than in LSL (*P* ≤ 0.010), higher at 30 than 42 weeks (*P* < 0.001), and higher when fed P+ than P− (*P* < 0.001). In the ileum, the InsP_6_ concentration was lower in LB fed P+ and in LSL fed P− than in other treatments (*P* = 0.027), while InsP_5_ isomer concentrations varied with age × strain interaction and age (*P* ≤ 0.021). The MI concentration was higher in LB hens fed P− than LSL hens and hens fed P+ (*P* ≤ 0.005). After *in vitro* incubation, the InsP_6_ concentration was higher with mucosa of 30-week-old hens fed P+ than in other treatments (*P* = 0.016). The InsP_6_ concentration after *in vitro* incubation was highly heritable only in LB ( $h^{2}$ = 0.62, *P* = 0.003) and a polygenic structure for this trait was detected. The consistent results from excreta, ileum digesta, and *in vitro* measures provide an extended view of endogenous mucosal phytate degradation primarily driven by a 6-phytase. Dietary P renunciation and aging of hens appeared to increase phytate degradation, possibly by increased expression of endogenous mucosal phosphatases. The differences between the two laying hen strains reveal different endogenous mechanisms and were reflected at the quantitative genetic level for the *in vitro* traits.

## 1 Introduction

The primary binding form of phosphorus (P) in plant feedstuff is phytate, the salt of *myo*-inositol hexakisphosphate (InsP_6_) (Eeckhout and De Paepe, 1994). Phytases and other phosphatases catalyze the hydrolysis of InsP_6_ in the digestive tract before InsP_6_-P is available for absorption. In this process, phytate is hydrolyzed into various inositol phosphate (InsP_x_) isomers with varying degrees of phosphorylation (InsP_1-5_) by releasing inorganic phosphate (P_i_). Upon complete dephosphorylation, *myo*-inositol (MI) is released. The enzymes in the digestive tract can have different origins: intrinsic phytases in the feed ingredients, exogenous phytases added to the feed, and endogenous phytases and other phosphatases produced by the epithelial cells or the microorganisms in the digestive tract. In non-ruminant animals, the endogenous phosphatase activity is variable and often insufficient, which leads to limitations in InsP_6_ degradation in the digestive tract (Rodehutscord et al., 2022).

In laying hens, only marginal total tract InsP_6_ disappearance was found when using cecectomized hens (Siegert et al., 2023). Studies on prececal InsP_6_ disappearance reported values of up to 47% (Van der Klis et al., 1997; Carlos and Edwards, 1998). The InsP_6_ disappearance and the concentrations of InsP isomers and MI in the digesta are influenced by several factors, such as the laying hen strain, which impacted the phytate-P utilization (Abudabos, 2012). The concentrations of InsP_6_ and InsP_5_ isomers in the digesta were consistently lower in Lohmann LSL-classic (LSL) hens than in Lohmann Brown-classic (LB) hens (Sommerfeld et al., 2020a; 2020b; 2024). Differences between the LB and LSL strains regarding population genetic structure and the genetic background of traits related to Ca and P metabolism were also notable in Haas et al. (revised manuscript submitted to Genetics, Selection and Evolution (GSE) for publication), who used data of the same experimental population as the present study. The age of the hens was another influencing factor for the concentration of InsP_5-6_ isomers in the jejunum and ileum (Sommerfeld et al., 2020a) and InsP_3-4_ in the terminal ileum (Sommerfeld et al., 2020a; 2024). However, the direction of these changes was inconsistent. It is well known that the extent of prececal InsP_6_ disappearance in broiler chickens is affected by dietary P and Ca supplements (Rodehutscord et al., 2022). In laying hens, such effects have not been studied extensively. Nevertheless, removing monocalcium phosphate (MCP) from the diet reduced InsP_3-5_ isomer concentrations in the excreta of cecectomized laying hens (Siegert et al., 2023). Moreover, feeding a diet without a mineral P supplement compared to a P-supplemented diet decreased the concentrations of InsP_6_ and InsP_5_ isomers in the small intestine of laying hens, indicating a faster and more extensive InsP degradation (Sommerfeld et al., 2024). Increased dietary Ca concentrations also adversely influenced InsP_6_ degradation in laying hens (Van der Klis et al., 1997). Sommerfeld et al. (2020b) reported that reducing dietary Ca concentration increased MI concentrations in the jejunum and ileum digesta. In a subsequent study, Sommerfeld et al. (2024) observed that higher digesta Ca concentrations in the duodenum + jejunum were associated with elevated concentrations of InsP_6_ and reduced concentrations of MI in the duodenum + jejunum.

Measurements of InsP_x_ concentrations in digesta and excreta obtained *in vivo* cannot distinguish between isomers produced by enzymes of the feed, endogenous microbes, and mucosa. Isolating the mucosa and using brush border membrane (BBM) preparations *in vitro* allowed for a distinct view on mucosal phosphatases and showed that the age of laying hens and the Ca concentration of the feed impacted these enzymes (Hanauska et al., 2025). Nevertheless, *in vivo* and *in vitro* measurements have not yet been combined for endogenous mucosal phosphatases characterization. As InsP_x_ concentrations *in vitro* explicitly represent the endogenous phosphatase activity of animals, these may provide a suitable basis to quantify genetic effects on this trait. Such knowledge is critical to infer if selective breeding could improve phosphatase activity and generate more profound insights into differences in InsP_6_ degradation between laying hen strains.

The availability of P is of considerable importance, as inorganic P sources are costly. Consequently, a major objective in poultry nutrition is to enhance the animal’s capacity to release phytate-bound P through endogenous enzymes. This is essential for achieving meaningful biological improvements in P utilization efficiency. Thus, the aim of the study was to investigate endogenous mucosal phosphatases by combining *in vivo* and *in vitro* measurements in order to evaluate phytate degradation products attributable to these specific enzymes and to determine the presence of an underlying quantitative genetic background. The main objective was to study InsP_x_ isomers in the excreta and terminal ileum, and to align these data with endogenous mucosal enzyme characterization in *in vitro* incubations using duodenal mucosa. The second objective was to compare two genetic strains of laying hens at two time points during laying peak when fed diets with or without mineral P supplementation. The third objective was to estimate genetic parameters for *in vitro* measures using mixed linear models. It was hypothesized that mineral P supplementation reduces *in vivo* degradation of InsP_6_ during laying peak and that this effect was substantiated by *in vitro* measures. The second hypothesis was that the laying hen strain and age influence hydrolysis products *in vivo* and *in vitro*. A third hypothesis proposed that the *in vitro* traits associate with quantitative genetic differences between the two strains.

## 2 Materials and methods

### 2.1 Animals and diets

This study was part of the interdisciplinary Research Unit P-Fowl–Inositol phosphates and *myo*-inositol in the domestic fowl: Exploring the interface of genetics, physiology, microbiome, and nutrition (https://p-fowl.uni-hohenheim.de/). The animal trial complied with the German animal welfare legislation following approval by the Regierungspräsidium Tübingen, Germany (Project no. HOH67-21 TE). The design of the study, which the present study was a part of, was described in detail by Haas et al. (revised manuscript submitted to GSE for publication). The complete study involved a total of four cohorts of hens at four time points during the peak of egg production (week 30–42 of age) and no mineral P supplementation of the feed. The present study used only material from the first (week 30) and fourth (week 42) cohort, and additionally included a control diet with supplemented mineral P. In brief, the present study followed a 2 × 2 × 2-factorial arrangement of treatments and involved the factors hen strain, hen age, and P concentration of the feed. Hens of the strain LB (n = 110) and LSL (n = 110) with defined family structure were placed individually in metabolic units when they were 27 and 39 weeks old. At each time point, a total of 55 hens per strain were assigned to one of two dietary treatments (without [P−] and with [P+] mineral P supplement). Of these, 50 hens received the P− diet and five hens the P+ diet, following a randomized design. This distribution followed the aim of having a sufficient number of birds fed the P− diet for genetic investigations (Haas et al., revised manuscript submitted to GSE for publication). The diets were corn and soybean meal-based to minimize plant intrinsic phytase activity, formulated to meet the supply recommendations for laying hens (Gesellschaft für Ernährungsphysiologie, 1999), except for P, and provided to the hens for *ad libitum* consumption (Table 1). Monocalcium phosphate was included at 1 g P/kg in the P+ diets. Both diets contained TiO_2_ as an indigestible marker. Exogenous phytase was not added. The calculated nutrient concentrations were confirmed by analysis (Table 2). One LB hen from the first period had to be removed during the trial, and no other losses occurred.

**TABLE 1:** Ingredient and calculated composition of experimental layer diets^a^.

Ingredients, g/kg	P−^b^	P+^c^
Corn	597.1	594.0
Soybean meal	256.0	256.0
Alfalfa meal	30.0	30.0
Soybean oil	15.0	15.0
DL-Methionine	4.5	4.5
L-Lysine sulphate	0.9	0.9
Monocalcium phosphate	0.0	4.8
Limestone, fine	25.2	24.5
Limestone, coarse	58.0	57.0
Sodium chloride	2.8	2.8
Choline chloride	1.0	1.0
Sodium bicarbonate	2.0	2.0
Vitamin mix^d^	2.0	2.0
Mineral mix^e^	0.5	0.5
TiO_2_	5.0	5.0

Calculated, g/kg		
P	3.0	4.0
NPP	1.2	2.2
Ca	35	35
CP	166	165
ME, MJ/kg	11.4	11.3

^a^ Originally published by Haas et al. (revised manuscript submitted to GSE for publication).

^b^ P− = without mineral P supplement.

^c^ P+ = with 1 g supplement P/kg.

^d^ Vitamin premix (Miavit GmbH, Essen, Germany), provided per kg of the complete diet: 10,000 IU, vitamin A, 3,000 IU, vitamin D3, 30 mg vitamin E, 2.4 mg vitamin K3, 100 mcg biotin, 1 mg folic acid, 3 mg vitamin B1, 6 mg vitamin B2, 6 mg vitamin B6, 30 mcg vitamin B12, 50 mg nicotinamide, 14 mg calcium-D-pantothene.

^e^ Trace element premix (Gelamin Gesellschaft für Tierernährung mbH, Memmingen, Germany), provided per kg of complete diet: 80 mg manganese from manganese-(II)-oxide, 60 mg zinc from zinc sulfate monohydrate, 25 mg iron from ferrous-(II)-sulfate monohydrate, 7.5 mg copper from cupric-(II)-sulfate pentahydrate, 0.6 mg iodine from calcium iodate, 0.2 mg selenium from sodium selenite.

**TABLE 2:** Analyzed composition of experimental layer diets^a^.

	P−^b^	P+^c^
	g/kg DM	
P	3.6	4.7
Ca	35.1	36.6
*Myo*-inositol	0.2	0.2
Ins(1,2,4,5,6)P_5_	0.4	0.4
InsP_6_	6.9	6.8
InsP_6_-P	1.9	1.9
Ti	3.0	3.2

	P−^b^	P+^c^
	*µ*mol/g DM^d^	
*Myo*-inositol	1.3	1.3
Ins(1,2,3,4,5)P_5_	<LOQ	<LOQ
Ins(1,2,4,5,6)P_5_	0.7	0.7
InsP_6_	10.5	10.3

^a^ Originally published by Haas et al. (revised manuscript submitted to GSE for publication).

^b^ P− = without mineral P supplement.

^c^ P+ = with 1 g supplement P/kg.

^d^ All other InsP_x_ isomers below the limit of quantification (LOQ) or limit of detection.

### 2.2 Experimental procedures, samplings, and measurements

Total excreta of individuals were collected at 24-h intervals over four consecutive days during the week preceding slaughter and subsequently frozen at −20 °C. Later, excreta samples were thawed at 4 °C, weighed, pooled for each hen, and homogenized. A subsample was freeze-dried (Delta 1–24 LSC, Martin Christ, Osterode, Germany), pulverized (PULVERISETTE 9, Fritsch GmbH, Idar-Oberstein, Germany), and stored in airtight containers until further analysis.

After a 3-week stay in the metabolism units, the hens were stunned using a gas mixture (35% CO_2_, 35% N_2_, 30% O_2_) and killed by exsanguination in weeks 30 and 42, respectively. Immediately after exsanguination, the duodenum was excised, opened longitudinally, and rinsed with cold physiological saline (0.9% NaCl). It was then carefully enrolled on a glass plate on ice. Mucosa from the entire section was stripped off from the muscle layer with microscopic slides and immediately shock-frozen in liquid nitrogen. The tubes were transported on dry ice to the laboratory and stored at −80 °C (HERAfreeze™ HFU T Series −86 °C Thermo Scientific™, Fisher Scientific GmbH, Schwerte, Germany) until further processing. Digesta sampling was conducted as described by Sommerfeld et al. (2024). Briefly, the terminal ileum, defined as the distal two-thirds of the segment between Meckel’s diverticulum and 2 cm proximal to the ileo-ceco-colonic junction, was excised by gentle squeezing, and the samples were immediately frozen at −20 °C. Digesta samples were freeze-dried and processed following the same procedures described for excreta samples. Feed samples were ground through a 0.5 mm sieve (Ultra Centrifugal Mill ZM 200, Retsch GmbH, Haan, Germany) and pulverized following the same procedure described for excreta samples.

### 2.3 Sample preparation and analyses

The pulverized feed, digesta, and excreta samples were analyzed for dry matter (DM) (method no. 3.1) and for P, Ca, and Ti by inductively coupled plasma-optical emission spectrometry following wet digestion using the method of Boguhn et al. (2009) with modifications by Zeller et al. (2015). The entire duodenum mucosa sample obtained from each hen was transferred from the 5-mL CryoPure tubes (CryoPure, 5 mL, QuickSeal cap, Sarstedt AG and Co. KG, Nümbrecht, Germany) into 10 mL aluminum cryogenic boxes (ROTILABO®, ROTH SELECTION, Carl Roth GmbH + Co. KG, Karlsruhe, Germany) and subsequently weighed. After 24 h of freeze-drying (Delta 1–24 LSC, Martin Christ, Osterode, Germany), the samples were weighed again and ground with a mortar and pestle. The protein content of the freeze-dried mucosa was quantified in triplicate using the Bradford assay (Bradford Reagent, five x, SERVA, Heidelberg, Germany).

### 2.4 InsP_6_ degradation assay

The three-step *in vitro* assay described by Hanauska et al. (2025) was adapted for freeze-dried mucosa. The assay was designed to simulate *in vivo* retention time, temperature, pH, and gastric and pancreatic enzymes of the crop, stomach, and small intestine in poultry with mucosa. The protein quantification assay was modified to standardize freeze-dried mucosa inclusion. Therefore, the freeze-dried mucosa powder was resuspended in 4-(2-hydroxyethyl)piperazine-1-ethanesulfonic acid/mannitol buffer (HEPES 2 mM, mannitol 50 mM, PMSF 25 mM). The required amount of freeze-dried mucosa corresponded to 0.025 g of fresh mucosa for 250 *µ*L of buffer. Previous tests indicated that optimal dissolution of the mucosa occurred after 5 h at room temperature without detectable protein degradation (data not shown). A calculated amount of freeze-dried mucosa was resuspended in 250 *µ*L buffer for 5 h and immediately diluted for protein quantification.

For the three-step *in vitro* assay, a corn and soybean meal mixture containing 10.8 *µ*mol InsP_6_/g, 0.6 *µ*mol Ins(1,2,4,5,6)P_5_/g, and 0.3 *µ*mol Ins(1,2,3,4,5)P_5_/g was used (Table 3). For the incubation, 0.1 g of the mixture was weighed into 10 mL tubes. Step 1 started with adding 150 *µ*L of double-distilled water, ensuring adequate moistening of the substrate, and 7.5 *µ*L of 0.25 M HCl to achieve a pH of 5.8. Two glass beads (Glass beads 5 mm, Merck KGaA, Darmstadt, Germany) were added and the tubes were vortexed. Samples were incubated at 40 °C for 30 min in a shaking water bath with a 20 L water chamber (Shaking Water Baths GFL-1083, GFL Gesellschaft für Labortechnik mbH, Burgwedel, Germany). Immediately after the initiation of step 1, the freeze-dried mucosa was mixed with double-distilled water to ensure that it remained in solution until the third step. Step 2 started with the addition of 5 *µ*L double-distilled water containing 300 U pepsin (from porcine gastric mucosa, 77,160-100G, Sigma-Aldrich, St. Louis, MO, United States) and 146 *µ*L 0.25 M HCl to achieve a pH of 2.8. Samples were vortexed and incubated again for 45 min at conditions as in step 1. In step 3, 38.5 *µ*L pancreatin solution (3.7 mg pancreatin/mL 1 M NaHCO_3_ (from porcine pancreas, P7545-25G, Sigma-Aldrich, St Louis, MO, United States)) were added to achieve a pH of 6.1. After vortexing, freeze-dried duodenum mucosa in solution, standardized at an amount equivalent to 5 mg freeze-dried duodenum mucosa protein, was added and the whole mix was vortexed again and incubated for 60 min. Previous test runs of this assay determined the amount of freeze-dried mucosa protein needed to ensure that the amount of InsP_6_ from the substrate was not a limiting factor for the enzymes (data not shown). The assay was run in duplicate with freeze-dried mucosa in solution from each of the 219 hens. Samples without mucosa addition were included as a baseline to distinguish between InsP_x_ produced by the mucosa enzymes and those possibly produced by traces of feed phytases.

**TABLE 3:** Analyzed concentrations of inositol phosphates in the feed ingredients and the mixture before and after incubation in the *in vitro* assay without using freeze-dried mucosa.

	InsP_3x_	Ins(1,2,5,6)P_4_	Ins(1,2,3,4,6)P_5_	Ins(1,2,3,4,5)P_5_	Ins(1,2,4,5,6)P_5_	InsP_6_
	*µ*mol/g					
Corn	n.d.^a^	n.d.	n.d.	<LOQ^b^	<LOQ	8.9
Soybean meal	<LOQ	<LOQ	<LOQ	0.8	1.4	15.8
Corn-soybean meal mixture	n.d.	n.d.	<LOQ	0.3	0.6	10.8
Incubation residue of corn-soybean meal mixture	n.d.	n.d.	<LOQ	0.3	0.6	10.5

^a^ n.d., not detectable (<0.1 *μ*mol/g).

^b^ <LOQ, below limit of quantification (0.3 *μ*mol/g).

Data are given as LSmeans; n = 3 for InsP_x_ analysis of the matrix and n = 9 for InsP_x_ analysis of the baseline.

### 2.5 Determination of InsP_x_ isomers and MI

The *in vitro* incubation was immediately followed by InsP_x_ extraction as described by Zeller et al. (2015) with slight modifications described by Sommerfeld et al. (2018). The InsP_x_ in feed, digesta, and excreta samples were also extracted using this method. High-performance ion chromatography (ICS-5000 system, Dionex, Thermo Scientific, Idstein, Germany) was used for InsP_3-6_ measurement. Separating InsP_x_ enantiomers and distinguishing D- and L-forms was impossible with this method. The isomers Ins(1,2,6)P_3_, Ins(1,4,5)P_3_, and Ins(2,4,5)P_3_ are referred to as InsP_3x_, as they could not be distinguished due to coelution. The fraction of InsP_x_ isomers lower than InsP_3x_ after *in vitro* incubation (herein referred to as InsP_<3_) was estimated by summing the concentrations of all measured InsP_x_ isomers in the baseline samples and subtracting the sum of all measured InsP_x_ isomers of each hen after incubation. The difference between these sums was considered as the InsP_<3_ amount. In the feed and digesta, MI was analyzed according to Sommerfeld et al. (2018) with gas chromatography/mass spectrometry after derivatization.

### 2.6 Calculations and statistical analysis

The InsP_6_ disappearance in the excreta was calculated based on the analyzed concentration of InsP_6_ and Ti in the feed and excreta. The following generally accepted equation was used (Equation 1):

$$Y\left({\text{InsP}}_{6}\right)=100-100\times \left(\frac{{\text{InsP}}_{6}\;\text{in}\;\text{excreta}\times \text{Ti}\;\text{in}\;\text{feed}\;\left(g/\text{kg}\;\text{DM}\right)\;}{\text{Ti}\;\text{in}\;\text{excreta}\;\left(g/\text{kg}\;\text{DM}\right)\times {\text{InsP}}_{6}\;\text{in}\;\text{feed}}\right)$$ (1)

where Y(InsP_6_) is the disappearance of InsP_6_ in %. The InsP_6_ disappearance after *in vitro* incubation was calculated using the InsP_6_ concentration in the corn-soybean meal mixture and the incubation residues.

Data from 50 hens per strain and age for P− and five hens per strain and age for P+ were analyzed with a 3-way ANOVA using the MIXED procedure of the software package SAS (Version 9.4, SAS Institute Inc., Cary, North Carolina). The following equation was used (Equation 2):

$$Y_{\text{ijklmn}}=\mu +\alpha_{i}+\beta_{j}+\gamma_{k}+{\left(\alpha \beta \right)}_{ij}+{\left(\alpha \gamma \right)}_{ik}+{\left(\beta \gamma \right)}_{ik}+{\left(\alpha \beta \gamma \right)}_{ijk}+\delta_{l}+\phi_{m}+\chi_{n}+\varepsilon_{\text{ijklmn}}$$ (2)

where Y_ijklmn_ = response variable, μ = overall mean, α_i_ = effect of hen strain (fixed), β_j_ = effect of hen age in weeks (fixed), γ_k_ = effect of dietary P (fixed), all two- and three-way-interactions among hen strain, hen age, and dietary P (fixed), δ_l_ = father/rooster (random), ϕ_m_ = slaughter day (random), χ_n_ = unit (random), and ε_ijklmn_ = residual error. The individual hen was considered as the experimental unit.

To further explore the inter-individual variation in InsP_6_ concentrations after *in vitro* incubation, an additional grouping was made across the three study factors. Hens were clustered based on the lowest (C_low_) and highest (C_high_) 15% of InsP_6_ concentrations after mucosa incubation (n = 33). A slightly modified version of Equation 2 was applied to assess whether hens of these clusters differ (Equation 3):

$$Y_{\text{ijkl}}=\mu +\alpha_{i}+\delta_{j}+\phi_{k}+\chi_{l}+\varepsilon_{\text{ijkl}}$$ (3)

where Y_ijkl_ = response variable, μ = overall mean, α_i_ = effect of high or low InsP_6_ concentration after *in vitro* incubation (fixed), δ_j_ = father/rooster (random), ϕ_k_ = slaughter day (random), χ_l_ = unit (random), and ε_ijkl_ = residual error. The individual hen was the experimental unit. Correlations were calculated using the CORR procedure of SAS. Statistical significance was set at *P* < 0.050. Regressions were calculated and results were visualized using GraphPad Prism (Version 5.0, GraphPad Software Inc., San Diego, CA, United States).

To quantify the additive genetic trait variation of the isomers generated *in vitro*, variance component analysis was conducted following Haas et al. (revised manuscript submitted to GSE for publication). As this study revealed large genetic differences between strains, all quantitative genetic analyses were separately performed in each of the strains. The hens that received the P− diet were considered for genetic analyses (n = 100 for LSL and n = 98 for LB) and were genotyped using a 50 k SNP-chip (Illumina, San Diego, CA). After quality filtering, 15,824 and 22,450 SNPs remained for genetic analyses in the LSL and LB strains. Since the phenotypes showed a highly skewed distribution, a Box-Cox transformation was applied for each trait. The variance component analysis was performed using the following mixed linear animal model in R (Version 4.2.3) (R Core Team, 2021) using ASReml-R (Version 4.1) (Butler et al., 2017) (Equation 4):

$$y=Xb+Z_{a}+Wsld+e$$ (4)

where y is the vector of Box-Cox transformed, scaled, and centered (mean = 0, SD = 1) phenotypes, b is the vector of the fixed effects cohort and weight of the egg in the oviduct as well as the number of intact, consumed and defect eggs (each of them included as a linear and squared regression variable), a is the vector of random animal effects, modeled as $a∼N\left(0,G\sigma_{a}^{2}\right)$ with $\sigma_{a}^{2}$ being the additive genetic variance, and G the additive genomic relationship matrix. Matrix G is modeled according to the first method described by VanRaden (2008), as $G=\frac{ZZ^{T}}{\sum_{j}2p_{j}\left(1-p_{j}\right)}$ , where Z is an n×m matrix with n the number of animals and m the number of centred genotypes, and $p_{j}$ is the allele frequency of the reference allele at SNP j . Vector sld models the random slaughter day effects as $sld∼N\left(0,I\sigma_{sld}^{2}\right)$ , where $\sigma_{sld}^{2}$ is the slaughter day variance and I the identity matrix. X denotes the design matrix for the fixed effects, $Z_{a}$ , and W denotes the corresponding incidence matrices for the respective random effects. Finally, vector e is the vector of random residuals, assumed to follow a normal distribution as $e∼N\left(0,I\sigma_{e}^{2}\right)$ , with residual variance $\sigma_{e}^{2}$ .

Heritability ( $h^{2}$ ) was calculated strain-wise as $h^{2}=\frac{\sigma_{a}^{2}}{\sigma_{a}^{2}+\sigma_{sld}^{2}+\sigma_{e}^{2}}$ by using the respective variance component estimates of LSL and LB hens. Significance of heritability was assessed by likelihood-ratio tests, testing whether the additive genetic variance differs significantly from zero.

Because genomic trait architecture refers to the SNP markers involved in trait expression and their effect sizes, SNP effects are of interest in inferring the genetic makeup of the traits. Therefore, the estimated SNP effects ( $\hat{u}$ ) were back-solved from the additive genetic animal effects ( $\hat{a}$ ) estimated with Model four by the following calculation: $\hat{u}=\frac{1}{m}Z^{T}G^{-1}\hat{a}$ . Such an evaluation was only valid for traits with a significant additive genetic variance component.

## 3 Results

The concentrations of InsP_x_ isomers in the excreta were not influenced by three- or two-way interactions among dietary P, hen strain, and hen age (Table 4). The concentrations of InsP_6_, Ins(1,2,3,4,5)P_5_, and Ins(1,2,3,4,6)P_5_ were significantly lower in LB hens than in LSL hens (*P* ≤ 0.010; Table 4; Supplementary Table S2). Correspondingly, the InsP_6_ disappearance was greater in LB hens than in LSL hens (*P* = 0.036). Hens fed P− excreted significantly less InsP_6_, Ins(1,2,4,5,6)P_5_, Ins(1,2,3,4,5)P_5_, and Ins(1,2,3,4,6)P_5_ compared to those fed P+ (*P* ≤ 0.003). In the excreta of the 42-week-old hens, the concentrations of InsP_6_, Ins(1,2,4,5,6)P_5_, and Ins(1,2,3,4,5)P_5_ were significantly lower than in those of the 30-week-old hens (*P* < 0.001), which corresponded to a 7.7 percentage points higher InsP_6_ disappearance in the older hens (*P* < 0.001). The highest total P content of the excreta was observed in 30-week-old LSL hens, while it was 1.8 g P/kg DM lower in excreta of 42-week-old LSL hens. LB hens exhibited intermediate levels (*P* < 0.001; Table 4; Supplementary Table S1).

**TABLE 4:** Effects of hen age, dietary P, and hen strain on concentrations of inositol phosphates and InsP_6_ disappearance in the excreta (P− treatment: n = 49–50 hens per strain and age and P+ treatment: n = 5 hens per strain and age; n = 219 hens in total). Two-way interactions and main effects are shown in Supplementary Tables S1, S2 of the supplement.

			Ins(1,2,3,4)P_4_	Ins(1,2,3,4,6)P_5_	Ins(1,2,3,4,5)P_5_	Ins(1,2,4,5,6)P_5_	InsP_6_	InsP_6_ disappearance	Ca	P
Hen age	Dietary P	Hen strain	*µ*mol/g					%	g/kg DM	
30	P−^a^	LB^b^	n.d	0.2	1.2	1.7	26.2	14.2	47.2	9.8
30	P−	LSL^c^	0.1	0.4	1.3	1.7	28.5	11.2	43.5	10.7
30	P+^d^	LB	n.d	0.4	1.3	2.4	27.6	14.5	51.5	13.1
30	P+	LSL	n.d	0.6	1.5	2.4	30.7	11.4	46.8	13.9
42	P−	LB	n.d	n.d	0.9	1.2	23.4	27.0	43.4	9.8
42	P−	LSL	n.d	n.d	1.1	1.2	25.2	19.6	42.7	9.5
42	P+	LB	n.d	n.d	1.1	1.7	26.1	19.9	45.3	13.0
42	P+	LSL	n.d	n.d	1.2	1.6	27.0	15.3	43.0	11.5
		Pooled SEM P−		0.03	0.02	0.05	0.43	1.30	2.15	0.13
		Pooled SEM P+		0.07	0.05	0.10	0.89	2.91	3.87	0.32
*P*-values	Age				<0.001	<0.001	<0.001	<0.001	0.300	<0.001
	Strain			0.010	<0.001	0.524	0.002	0.036	0.238	0.887
	P			0.003	<0.001	<0.001	<0.001	0.143	0.307	<0.001
	Strain × P			0.950	0.997	0.639	0.933	0.726	0.791	0.140
	Age × P				0.311	0.083	0.712	0.117	0.566	0.122
	Age × strain				0.438	0.764	0.258	0.434	0.572	<0.001
	Age × strain × P				0.184	0.647	0.458	0.711	0.948	0.290

^a^ P− = without mineral P supplement.

^b^ LB, Lohmann Brown-classic.

^c^ LSL, Lohmann LSL-classic.

^d^ P+ = with 1 g supplement P/kg.

Data are given as LSmeans.

The ileal concentrations of MI and InsP_x_ isomers were unaffected by three-way interactions of dietary P, hen strain, and age (Table 5). The ileal concentrations of InsP_6_ and Ins(1,2,4,5,6)P_5_ were higher in 30-week-old LB and 42-week-old LSL hens than in 30-week-old LSL and 42-week-old LB hens (*P* ≤ 0.049; Table 5; Supplementary Table S3). The highest InsP_6_ concentrations were found in LB hens fed P− and the lowest in LB hens fed P+. LSL hens exhibited intermediate values with lower concentrations when fed P− (Supplementary Table S3). The Ins(1,2,3,4,5)P_5_ concentration in the ileum was significantly higher for 42-week-old hens than for 30-week-old hens (*P* = 0.021; Table 5; Supplementary Table S4). The concentration of InsP_6_ in the ileum was numerically higher for P−, while that of Ins(1,2,4,5,6)P_5_ was significantly reduced (*P* = 0.018) for P− compared to P+. The Ins(1,2,3,4,5)P_5_ concentration remained at a similar level in both treatments and Ins(1,2,3,4,6)P_5_ was detectable only for P−. The ileal MI concentration was 0.5 *μ*mol/g DM greater in LB hens than in LSL hens (*P* = 0.002) and 1.0 *µ*mol greater for P− than for P+ (*P* = 0.005).

**TABLE 5:** Effects of hen age, dietary P, and hen strain on concentrations of inositol phosphates and MI in the ileum digesta (P− treatment: n = 49–50 hens per strain and age and P+ treatment: n = 5 hens per strain and age; n = 219 hens in total). Two-way interactions and main effects are shown in Supplementary Tables S3, S4 of the supplement.

			MI^a^	Ins(1,2,3,4,6)P_5_	Ins(1,2,3,4,5)P_5_	Ins(1,2,4,5,6)P_5_	InsP_6_
Hen age	Dietary P	Hen strain	*µ*mol/g DM				
30	P−^b^	LB^c^	3.5	0.3	1.1	1.8	36.5
30	P−	LSL^d^	2.0	0.3	1.0	1.4	31.7
30	P+^e^	LB	3.1	0.3	1.1	2.1	31.1
30	P+	LSL	1.1	n.d	1.0	1.6	30.8
42	P−	LB	4.3	0.5	1.2	1.6	33.0
42	P−	LSL	2.3	0.5	1.2	1.6	31.6
42	P+	LB	2.2	0.3	1.0	1.6	27.5
42	P+	LSL	1.7	0.6	1.4	2.2	37.8
		Pooled SEM P−	0.33	0.03	0.05	0.10	1.35
		Pooled SEM P+	0.69	0.07	0.12	0.20	2.99
*P*-values	Age		0.628	0.150	0.021	0.786	0.983
	Strain		0.002	0.032	0.288	0.406	0.591
	P		0.005	0.432	0.920	0.018	0.436
	Strain × P		0.511	0.022	0.128	0.333	0.027
	Age × P		0.368	0.093	0.716	0.956	0.326
	Age × strain		0.548	0.892	0.072	0.005	0.049
	Age × strain × P		0.166		0.297	0.130	0.313

^a^ MI, *myo*-inositol.

^b^ P− = without mineral P supplement.

^c^ LB, Lohmann Brown-classic.

^d^ LSL, Lohmann LSL-classic.

^e^ P+ = with 1 g supplement P/kg.

Data are given as LSmeans.

After incubation with mucosa, the InsP_6_ disappearance and InsP_x_ isomers were unaffected by three-way interactions of dietary P, hen strain, and hen age (Table 6). InsP_6_ disappearance values after mucosa incubation were similar between both ages when fed P− but reduced when fed P+ by 15% with mucosa of 30-week-old hens and by 6.5% with mucosa of 42-week-old hens (*P* = 0.016; Table 6; Supplementary Table S5). The concentrations of Ins(1,2,3,4,5)P_5_ and InsP_<3_ were 0.3 *μ*mol/g greater and 0.9 *μ*mol/g smaller for P+ compared to P−, respectively (*P* ≤ 0.001; Table 6; Supplementary Table S6). The highest concentration of Ins(1,2,3,4)P_4_ was observed with mucosa of LSL hens fed P+ compared to the other groups (*P* = 0.015; Table 6; Supplementary Table S5). InsP_3x_ concentrations were higher for P− than P+ and higher in LSL hens than in LB hens (*P* = 0.042; Table 6; Supplementary Table S6). The coefficient of variation (CV%) of InsP_6_ concentration in the incubation residue varied between 0.0% and 11.1% in samples incubated with mucosa, with a mean of 3.0%. In samples incubated without mucosa, the CV% ranged from 0.0% to 7.2%, averaging 2.4%.

**TABLE 6:** Effects of hen age, dietary P, and hen strain on concentrations of inositol phosphates in the incubation residue and InsP_6_ disappearance in the three-step *in vitro* assay using freeze-dried mucosa of laying hens (P− treatment: n = 49–50 hens per strain and age and P+ treatment: n = 5 hens per strain and age; n = 219 hens in total). Two-way interactions and main effects are shown in Supplementary Tables S5, S6 of the supplement.

			InsP_3x_ ^a^	Ins(1,2,3,4) P_4_	Ins(1,2,3,4,6)P_5_	Ins(1,2,3,4,5)P_5_	Ins(1,2,4,5,6)P_5_	InsP_6_	InsP_6_ disappearance	InsP_<3_ ^b^
Hen age	Dietary P	Hen strain	*µ*mol/g						%	*µ*mol/g
30	P−^c^	LB^d^	2.4	1.3	<LOQ^e^	0.4	n.d^f^	5.0	53.7	2.5
30	P−	LSL^g^	2.6	1.4	<LOQ	0.4	n.d	5.0	54.3	2.5
30	P+^h^	LB	1.1	1.1	<LOQ	0.8	0.2	7.0	36.0	1.3
30	P+	LSL	2.1	1.6	<LOQ	0.7	<LOQ	6.2	42.7	1.3
42	P−	LB	2.5	1.5	<LOQ	0.5	<LOQ	5.0	54.0	2.3
42	P−	LSL	2.7	1.6	<LOQ	0.4	<LOQ	4.9	55.3	2.3
42	P+	LB	1.8	1.5	<LOQ	0.7	<LOQ	5.8	46.8	1.9
42	P+	LSL	2.1	1.7	<LOQ	0.6	<LOQ	5.6	48.7	1.6
		Pooled SEM P−	0.09	0.05		0.03		0.14	1.34	0.14
		Pooled SEM P+	0.16	0.11		0.07		0.28	2.57	0.32
*P*-values	Age		0.112	0.023		0.959		0.033	0.036	0.547
	Strain		0.001	0.004		0.223		0.152	0.161	0.745
	P		<0.001	0.551		<0.001		<0.001	<0.001	<0.001
	Strain × P		0.042	0.015		0.375		0.294	0.295	0.675
	Age × P		0.243	0.810		0.291		0.016	0.016	0.148
	Age × strain		0.101	0.324		0.735		0.544	0.523	0.685
	Age × strain × P		0.097	0.427		0.863		0.409	0.387	0.754

^a^ InsP_3x_ = Ins(1,2,6)P_3_, Ins(1,4,5)P_3_, and Ins(2,4,5)P_3_ could not be differentiated due to co-elution and are thus referred to as InsP_3x_.

^b^ InsP_<3_ = fraction of InsP_x_ isomers lower than InsP_3x_ after *in vitro* incubation.

^c^ P− = without mineral P supplement.

^d^ LB, Lohmann Brown-classic.

^e^ <LOQ, below limit of quantification (for Ins(1,2,5,6)P_4_ and Ins(1,2,3,4,6)P_5_ 0.3 *μ*mol/g and for Ins(1,2,4,5,6)P_5_ 0.2 *μ*mol/g).

^f^ n.d. = not detectable (<0.1 *μ*mol/g).

^g^ LSL, Lohmann LSL-classic.

^h^ P+ = with 1 g supplement P/kg.

Data are given as LSmeans.

Values calculated for C_high_ and C_low_ clusters are shown in Table 7. The concentrations of Ins(1,2,3,4,6)P_5_, Ins(1,2,3,4,5)P_5_, Ins(1,2,4,5,6)P_5_, and P in the excreta were significantly lower in C_low_ compared to C_high_ (*P* ≤ 0.046). Similarly, hens in C_low_ exhibited a significantly higher ileal MI concentration (*P* = 0.050), while ileal concentrations of Ins(1,2,3,4,5)P_5,_ Ins(1,2,4,5,6)P_5_, and P were significantly lower compared to C_high_ (*P* ≤ 0.005). Following mucosa incubation, the concentration of InsP_<3x_ and InsP_3x_ were significantly higher (*P* ≤ 0.001), whereas concentrations of Ins(1,2,3,4)P_4,_ Ins(1,2,3,4,5)P_5_, and InsP_6_ were significantly lower (*P* ≤ 0.001) in C_low_ than in C_high_.

**TABLE 7:** Treatment distribution of hens representing the lowest and highest 15% of *in vitro* InsP_6_ concentrations (n = 33) and comparison of the *in vivo* and *in vitro* traits related to InsP_x_ degradation. 15% of hens with the highest (C_high_) and lowest (C_low_) *in vitro* InsP_6_ concentrations, irrespective of dietary P, hen strain, or hen age.

Variable	C_low_ (n = 33)	C_high_ (n = 33)	Pooled SEM	*P*-value
Treatment distribution	%	%		
P−^a^	100.0	60.6		
P+^b^	0.0	39.4		
LB^c^	48.5	57.6		
LSL^d^	51.5	42.4		
30-week-old	48.5	63.6		
42-week-old	51.5	36.4		
Ileum				
*Myo*-inositol (*µ*mol/g DM)	3.4	2.5	0.46	0.050
Ins(1,2,3,4,6)P_5_ (*µ*mol/g DM)	0.3	0.4	0.05	0.624
Ins(1,2,3,4,5)P_5_ (*µ*mol/g DM)	0.9	1.2	0.05	0.005
Ins(1,2,4,5,6)P_5_ (*µ*mol/g DM)	1.3	1.9	0.09	<0.001
InsP_6_ (*µ*mol/g DM)	29.9	32.8	1.42	0.143
Ca (g/kg DM)	36.3	41.3	4.06	0.407
P (g/kg DM)	9.2	10.6	0.33	0.004
Excreta				
Ins(1,2,3,4)P_4_ (*µ*mol/g DM)	0.2	0.1	0.04	0.331
Ins(1,2,3,4,6)P_5_ (*µ*mol/g DM)	0.3	0.4	0.05	0.046
Ins(1,2,3,4,5)P_5_ (*µ*mol/g DM)	1.1	1.2	0.07	0.002
Ins(1,2,4,5,6)P_5_ (*µ*mol/g DM)	1.3	1.8	0.13	<0.001
InsP_6_ (*µ*mol/g DM)	25.1	26.6	0.86	0.048
InsP_6_ disappearance (%)	20.8	15.4	2.59	0.031
Ca (g/kg DM)	43.5	48.8	2.64	0.074
P (g/kg DM)	9.9	11.0	0.31	0.007
*In vitro*				
InsP_<3x_ (*µ*mol/g)^e^	3.9	1.5	0.09	<0.001
InsP_3x_ (*µ*mol/g)^f^	2.7	1.5	0.08	<0.001
Ins(1,2,3,4)P_4_ (*µ*mol/g)	1.1	1.4	0.06	<0.001
Ins(1,2,3,4,5)P_5_ (*µ*mol/g)	0.2	0.7	0.02	<0.001
Ins(1,2,4,5,6)P_5_ (*µ*mol/g)	n.d	0.2		
InsP_6_ (*µ*mol/g)	3.9	6.5	0.08	<0.001
InsP_6_ disappearance (%)	64.1	40.1	0.78	<0.001

^a^ P− = without mineral P supplement.

^b^ P+ = with 1 g supplement P/kg.

^c^ LB, Lohmann Brown-classic.

^d^ LSL, Lohmann LSL-classic.

^e^ InsP_<3_ = fraction of InsP_x_ isomers lower than InsP_3x_ after *in vitro* incubation.

^f^ InsP_3x_ = Ins(1,2,6)P_3_, Ins(1,4,5)P_3_, and Ins(2,4,5)P_3_ could not be differentiated due to co-elution and are thus referred to as InsP_3x_.

Data are given as LSmeans.

The results of the variance component analyses of the *in vitro* traits of phytate degradation by duodenum mucosal phosphatases are displayed in Table 8. Generally, the estimated values differed greatly between LSL and LB hens. Except for Ins(1,2,3,4)P_4_, additive genetic variances were estimable in both strains, whereby the additive genetic variation was obviously larger in the LB than the LSL strain. High and significant *h* ^*2*^ (*P* < 0.050) was only found for the InsP_6_ concentration and InsP_6_ disappearance (*h* ^*2*^ = 0.62 and 0.59, respectively) in the LB strain, albeit with large standard errors.

**TABLE 8:** Results of the variance component estimation for the Lohmann LSL-classic (LSL) and Lohmann Brown-classic (LB) hens. The heritability (*h* ^*2*^), its *P* value, additive genetic variance (V_A_), slaughter day variance (V_SLD_), and residual variance (V_E_) for the *in vitro* measured traits. Standard errors (SE) are given in parentheses and significant (*P* < 0.050) heritabilities are highlighted in bold.

Trait	LSL (n = 100)					LB (n = 98)				
	*h* ^*2*^ (SE)	*P*-value	V_A_ (SE)	V_SLD_ (SE)	V_E_ (SE)	*h* ^*2*^ (SE)	*P*-value	V_A_ (SE)	V_SLD_ (SE)	V_E_ (SE)
InsP_<3_ (*µ*mol/g)	0.14 (0.25)	0.546	0.15 (0.24)	0.02 (0.07)	0.87 (0.26)	0.32 (0.29)	0.163	0.27 (0.23)	0.02 (0.06)	0.55 (0.21)
InsP_3x_ (*µ*mol/g)	0.05 (0.19)	0.787	0.05 (0.18)	0.04 (0.09)	0.88 (0.22)	0.24 (0.25)	0.133	0.20 (0.18)	n.e.*	0.62 (0.19)
Ins(1,2,3,4)P_4_ (*µ*mol/g)	0.02 (0.17)	0.883	0.02 (0.16)	0.06 (0.09)	0.87 (0.21)	n.e.*	n.e.*	n.e.*	0.08 (0.10)	0.75 (0.17)
InsP_6_ (*µ*mol/g)	0.06 (0.22)	0.817	0.06 (0.22)	0.06 (0.11)	0.90 (0.25)	**0.62 (0.30)**	**0.003**	0.41 (0.21)	n.e.*	0.25 (0.16)
InsP_6_ disappearance (%)	0.08 (0.23)	0.782	0.08 (0.22)	0.06 (0.10)	0.89 (0.25)	**0.59 (0.30)**	**0.004**	0.39 (0.21)	n.e.*	0.27 (0.16)

*not estimable.

The results of the back-solved SNP were characterized by small effect sizes, distributed across the entire genome with no prominent peak at any genomic region. This was observed for all traits and suggested a polygenic nature of the traits. These results are not shown for the sake of brevity.

## 4 Discussion

This study aimed to investigate the endogenous mucosal phosphatases in laying hens by analyzing InsP_x_ isomers in the excreta and the terminal ileum in combination with *in vitro* incubation of duodenal mucosa to evaluate the degradation products attributable to endogenous mucosal phosphatases specifically. For the first time, this study investigated the quantitative genetic background of *in vitro* traits in two laying hen strains. Comparing two different strains of laying hens at two time points during laying peak and when fed with or without mineral P supplementation provides novel insights into the role of endogenous mucosal phosphatases on InsP_x_ degradation, offering a complex perspective on nutrient utilization. The unequal number of P+ and P− fed hens in the present study resulted from the experimental design specifically tailored to enable genetic analyses of P− fed hens. The P+ fed hens were included only in smaller numbers as a reference in accordance with the 3Rs principle (reduce, replace, refine). Duodenal mucosa samples were chosen because this site exhibits the highest enzyme activity (Maenz and Classen, 1998) and represents the main site of the degradation processes under investigation. In contrast, ileal digesta samples were analyzed for degradation products, as this section reflects the entirety of prececal processes.

### 4.1 Effect of mineral P renunciation

**FIGURE 1:**
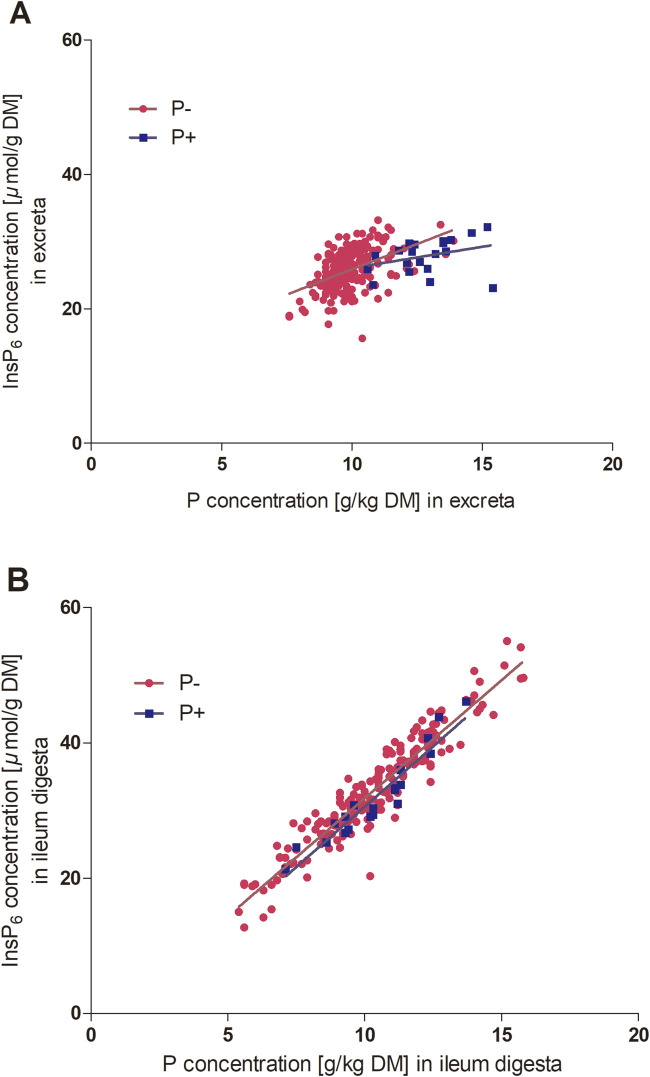
**(A)** Excreta P concentration (g/kg DM) and InsP_6_ concentration (*µ*mol/g DM) (P− n = 198; P+ n = 20 hens, 218 in total). P− y = 1.51 x + 10.82, *r* ^2^ = 0.23, *P* < 0.001 and P+ y = 0.63 x + 19.71, *r* ^2^ = 0.11, *P* = 0.155. **(B)** Ileum digesta P concentration (g/kg DM) and InsP_6_ concentration (*µ*mol/g DM) (P− n = 195; P+ n = 20 hens, 215 in total). P− y = 3.49 x – 3.10, *r* ^2^ = 0.89, *P* < 0.001 and P+ y = 3.57 x – 5.12, *r* ^2^ = 0.91, *P* < 0.001. The symbols indicate data points of individual hens (GraphPad Prism 5.0).

**FIGURE 2:**
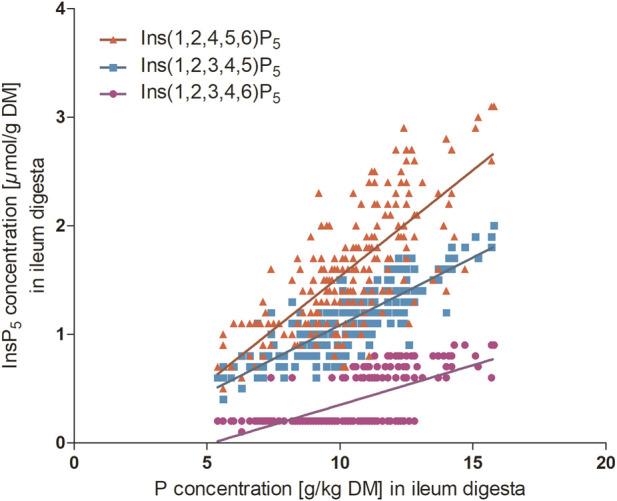
Ileal digesta P concentration (g/kg DM) and InsP_5_ isomer concentration (*µ*mol/g DM) of P+ and P– together. The symbols indicate data points of individual hens (Ins(1,2,4,5,6)P_5_ n = 215; Ins(1,2,3,4,5)P_5_ n = 215; Ins(1,2,3,4,6)P_5_ n = 210 hens; GraphPad Prism 5.0). Ins(1,2,4,5,6)P_5_ y = 0.20 x – 0.42, *r* ^2^ = 0.55, *P* < 0.001 and Ins(1,2,3,4,5)P_5_ y = 0.12 x – 0.16, *r* ^2^ = 0.70, *P* < 0.001 and Ins(1,2,3,4,6)P_5_ y = 0.07 x – 0.38, *r* ^2^ = 0.40, *P* < 0.001.

The initial hypothesis of enhanced InsP_6_ degradation due to renunciation of mineral dietary P was partially confirmed. InsP_6_ concentrations were lower when the hens were fed P− than P+ in the excreta, but not in the ileal content. This contradicts findings by Sommerfeld et al. (2024), who demonstrated lower concentrations of InsP_6_ in small intestine digesta of laying hens of the same strains fed a diet without mineral P compared to a diet with mineral P supplementation. As the diets in Sommerfeld et al. (2024) and the present study were almost identical, these discrepancies may be attributed to the different ages of the laying hens, covering the transition period from pullet (19-week-old) to laying hen (24-week-old) in Sommerfeld et al. (2024) and the peak of egg production in the present study. Furthermore, hens receiving the P− diet exhibited lower concentrations of InsP_5_ isomers in the excreta and reduced levels of Ins(1,2,4,5,6)P_5_ in the ileum. In contrast, concentrations of Ins(1,2,3,4,6)P_5_ and MI in the ileum were elevated, compared to hens fed P+. This may be explained by potential end product inhibition of mucosal phosphatases by mineral P, as previously described by Davies et al. (1970), who examined alkaline phosphatase and phytase activity in the proximal duodenal mucosa of chicks. In that study, enzyme activity was higher with lower P concentration in the feed, indicating that increased P availability may suppress mucosal phosphatase activity. The relationship between dietary P or ileal P concentrations and InsP_6_ hydrolysis has been previously reported in laying hens (Sommerfeld et al., 2024) and broiler chickens (Rodehutscord et al., 2022). Appropriately, positive correlations were observed between the concentrations of P and InsP_6_ (InsP_6_: r = 0.473; *P* ≤ 0.001) (Figure 1A) and between the concentrations of P and InsP_5_ isomers in the excreta (Ins(1,2,4,5,6)P_5_: r = 0.516; *P* ≤ 0.001; Ins(1,2,3,4,5)P_5_: r = 0.475; *P* ≤ 0.001; Ins(1,2,3,4,6)P_5_: r = 0.492; *P* ≤ 0.001). Additionally, positive correlations were observed between concentrations of ileal P and InsP_6_ (Figure 1B) and InsP_5_ isomers (Figure 2). The excreta data align with previously reported small intestine results (Sommerfeld et al., 2024) and the InsP_6_ concentrations after incubation with mucosa in the present study. In contrast, the present ileum InsP_6_ results appear inconsistent with the excreta and *in vitro* data. This discrepancy may be attributed to the fact that the comparison involves different sampling types–excreta, ileum digesta, and duodenum mucosa–characterized by different timing of sample collection and preparation (Zhai et al., 2020; Siegert et al., 2021). Ileal data represent a single sampling time point specific to each hen. This is particularly relevant in laying hens, whose circadian rhythm, driven by oviposition, modulates the hormonal rhythms and influences Ca and P metabolism (Frost et al., 1991; Reyer et al., 2021). In contrast, excreta data originate from pooled samples collected over four consecutive days, offering an integrated measure of nutrient excretion. However, microbial activity in the ceca may influence InsP_x_ degradation measured in excreta samples. In laying hens, the ceca exhibit the greatest microbial diversity within the gastrointestinal tract (Schokker et al., 2021; Roth et al., 2022), which could facilitate additional phytate hydrolysis by microbial enzymes. It is important to note that only a limited proportion of the ileal digesta enters the ceca, primarily the liquid phase and small particles, accounting for approximately 30%–50% of the total intestinal content (Vergara et al., 1989; Garçon et al., 2023). In laying hens, digesta retention time in the ceca ranges from 12 to 20 h (Schokker et al., 2021). Habibi et al. (2024) reported that solid digesta is retained for approximately 0.5 h, whereas liquid fractions remain for 2–5 h, with cecal emptying occurring a few times daily (Svihus et al., 2013). Considering their limited involvement in digesta passage, and the relatively low differences between ileum and excreta isomer concentrations in the present study, the ceca can be assumed to play a minor role in total gastrointestinal phytate degradation.

### 4.2 Effect of laying hen strain and hen age

The hypothesis regarding differences in InsP_x_ degradation between LB and LSL hens was confirmed. LB hens exhibited lower InsP_6,_ Ins(1,2,3,4,6)P_5_, and Ins(1,2,3,4,5)P_5_ concentrations in the excreta than LSL hens, which was also reflected by a lower InsP_x_ concentration after incubation with mucosa from LB than LSL hens. It has been suggested that LB hens exhibit an enhanced capacity for phytate degradation due to a potential higher physiological demand for P, which they may meet through the upregulation of genes involved in ileal transcellular P transport and intracellular InsP metabolism (Sommerfeld et al., 2020b; Sommerfeld et al., 2024).

Differences in InsP_6_ degradation between the strains were also found at the quantitative genetic level. This is consistent with the results of Haas et al. (revised manuscript submitted to GSE for publication), who found additive genetic variance for P-related traits mainly in the LB strain and hardly in the LSL strain. In the present study, genetic analysis focused on the *in vitro* concentrations of InsP_6_ and its degradation products, i.e., InsP_x_ isomers, after mucosa incubation. The *in vitro* approach implies that the endogenous mucosa is the only source of phosphatases active in the intestine (Hanauska et al., 2025). Hence, the *in vitro* results were particularly useful in shedding light on the genetic potential of animals to degrade InsP_6_. The additive genetic variation of ileal InsP_6_ concentration *in vivo* was negligibly small in the study of Haas et al. (revised manuscript submitted to GSE for publication), but the present study revealed high and significant heritability for *in vitro* measured InsP_6_, indicating that *in vitro*-generated data provided improved phenotypes for the genetic analyses without environmental effects such as feed or host-specific microbes. Notably, the standard error of the additive genetic variance component was high, which was also valid for the other trait with significant heritable variation, InsP_6_ disappearance. A validation in other datasets with larger sample sizes is required before bringing forward such *in vitro* approaches as a suitable tool for deep phenotyping P metabolism traits. A larger study power might also have improved the results of InsP_3x_ and InsP_<3_, where the genetic contribution to trait variance is obviously present, but can only be seen as a trend due to the lack of statistical significance.

The results of the back-solving method did not indicate any prominent SNP, which suggests a polygenic background of all investigated traits. However, the small number of animals per strain (n = 98 LB hens; n = 100 LSL hens) and relatively low SNP marker density may have compromised the power and precision of genome-wide associations in this study and hence the ability to identify genomic regions significantly associated with InsP_6_ degradation.

Significant age × strain effects on ileal InsP_x_ concentrations confirmed the effect of hen age on InsP_x_ degradation. The ileal InsP_6_ and Ins(1,2,4,5,6)P_5_ concentrations decreased in LB hens and increased in LSL hens from week 30 to 42. This indicates a higher degree of InsP_x_ hydrolysis and a more effective downstream degradation by the terminal ileum for the 30-week-old LSL hens and 42-week-old LB hens compared to the respective other weeks. A similar trend was reported by Sommerfeld et al. (2020a) from a study that compared 30- and 60-week-old hens. It is conceivable that both strains undergo age-related changes between weeks 30 and 42, but in opposite directions. An age effect was also observed in excreta and incubations with mucosa, with 30-week-old hens showing higher InsP_x_ concentrations in the excreta and higher Ins(1,2,3,4)P_4_ concentrations after incubation with mucosa than 42-week-old hens, indicating a reduced InsP_6_ degradation capacity in younger hens. In addition, InsP_6_ concentrations after incubation with mucosa were significantly affected by the age × diet interaction, with feeding P+ resulting in significant differences between 30- and 42-week-old hens. Although enzymatic activity was not assessed in the present study, the observed reduction in InsP_6_ degradation indicates lower phosphatase activity in the younger hens, consistent with findings by Hanauska et al. (2025).

### 4.3 Comparison of C_high_ and C_low_

The large number of individuals allowed a categorization of hens based on the residual InsP_6_ concentrations following *in vitro* incubation with mucosa, enabling the investigation of potential differences in further traits. All 33 hens of C_low_, having the lowest residual InsP_6_ concentration after incubation with mucosa, were associated with P−, with 49% representing the LB hens and 49% being 30-week-old. Thus, neither age nor strain appeared to influence InsP_6_ degradation with mucosa in this cluster, whereas the dietary P level did. Moreover, hens of C_low_ generally demonstrated higher InsP_x_ degradation in the excreta, ileum, and after incubation with mucosa. Furthermore, C_low_ hens showed reduced P concentrations in excreta and ileum digesta, while Ca concentrations in excreta and ileum remained unaffected. This variation is possibly attributed to differences in the capacities of endogenous phosphatases among individual laying hens. These C_low_ hens, due to exhibiting lower P concentrations in the excreta and ileum digesta, may have had an increased physiological demand for P, potentially resulting in upregulated endogenous phosphatase and P absorption. Conversely, hens of C_high_ may have exhibited a lower P requirement. Among the 33 hens of C_high_, 61% were fed P−, 58% belonged to the LB strain, and 64% were 30-week-old. In this cluster, reduced enzymatic degradation of phytate and intermediates likely resulted in elevated InsP_x_ concentrations. This supports the view that the individual impacted InsP_6_ degradation capacity after mucosa incubation, rather than only mineral P in the feed.

### 4.4 *In vivo* and *in vitro* InsP_6_ degradation assay with freeze-dried mucosa

Consistent with other studies (Sommerfeld et al., 2020b; Sommerfeld et al., 2024), ileal InsP_6_ concentration in laying hens was overall high in the present study. However, specific InsP_x_ isomers were measured in all samples not detected in the feed. The degradation pattern of InsP_x_ isomers is unique for each phosphatase and follows a defined pathway (Greiner et al., 1993). The InsP_x_ isomer patterns in excreta, ileum digesta, and after mucosa incubation resemble those reported by Sommerfeld et al. (2020a) and Hanauska et al. (2025), indicating predominantly 6-phytase and secondary 5-phytase activity in the duodenal mucosa of laying hens. Although the overall InsP_x_ pattern, defined by the presence of specific isomers, appeared comparable, the *in vivo* isomer concentrations differed markedly from those obtained after incubation with mucosa. Thus, the *in vitro* system might amplify InsP_x_ degradation, leading to the detection of InsP_4_ and InsP_3_ isomers that may not accumulate to detectable levels *in vivo*. This suggests that these isomers might also be generated *in vivo* in the small intestinal digesta but remain undetectable due to low concentrations or rapid further degradation by other phosphatases, potentially falling below the sensitivity threshold of the analytical method employed.

The type and amount of mucosal protein used *in vitro* affected the observed InsP_6_ degradation. The use of resuspended freeze-dried mucosa instead of enriched BBM, as in Hanauska et al. (2025), requires a more than three times higher amount of mucosal protein (5 mg freeze-dried mucosa protein) to achieve *in vitro* InsP_6_ degradation levels comparable with those obtained with BBM (1.6 mg BBM protein (Hanauska et al., 2025)). On the other hand, preparing freeze-dried material is less labor-intensive, making it a feasible alternative when sufficient mucosal tissue is available. This also suggests that either the freeze-drying process partially inactivates phosphatase activity, or the BBM preparation selectively enriches for enzymatically active proteins by effectively removing non-functional protein fractions. According to Meijer et al. (1977) and Nosworthy et al. (2009), freeze-drying does not affect enzyme activity, which supports the effectiveness of the BBM enrichment procedure, as less protein is required for incubations with BBM. This enrichment could be further verified by Western blot analysis of *β*-actin expression in the homogenates before BBM enrichment and the final BBM samples (Huber et al., 2015). Moreover, the present *in vitro* assay showed an average CV% of 3.0% with a standard deviation of 2.5%, indicating a robust reproducibility when using resuspended freeze-dried duodenum mucosa. Furthermore, the detection of InsP_x_ isomers similar to those reported by Hanauska et al. (2025) suggests that hydrolysis was mainly driven by endogenous mucosal phosphatases in freeze-dried duodenal mucosa, even though microbial phosphatases were not explicitly excluded, unlike in the study of Hanauska et al. (2025) using enriched BBM. For the differentiation between enzymes of the feed and mucosal phosphatases, baseline incubations were conducted without the addition of freeze-dried mucosa. Identical amounts of Ins(1,2,4,5,6)P_5_ and Ins(1,2,3,4,5)P_5_ were detected before and after the *in vitro* incubation, indicating no further degradation of the InsP_x_ isomers occurred, suggesting that the corn-soybean mixture did not contain any phosphatases.

## 5 Conclusion

The consistent InsP_x_ isomer patterns in excreta, ileum digesta, and incubations with mucosa provide an extensive view of phytate degradation by phosphatases of laying hens. The results indicate that InsP_6_ degradation is primarily mediated by 6-phytase and secondarily by 5-phytase, confirming that *in vitro* measurements are suitable for assessing endogenous mucosal phosphatases. Under the conditions of this study, endogenous mucosal phosphatase expression was enhanced with dietary P renunciation, and its capacity increased with age. Strain-specific differences revealed distinct endogenous mechanisms, further supported by quantitative genetic analysis. The absence of InsP_x_ isomers lower phosphorylated than InsP_5_ in ileal digesta, despite their formation *in vitro*, underscores the complexity of *in vivo* conditions. Future studies should investigate the heritability of *in vitro* traits using larger sample sizes to advanced genetic selection to reduce InsP_x_ isomers and enable deep phenotyping of P physiology.

## Data Availability

The original contributions presented in the study are included in the article/Supplementary Material, further inquiries can be directed to the corresponding author.
