# Supplementary material for: Genetic, age, and diet effects on phytate degradation of laying hens studied in combined in vivo and in vitro assays
Source: Front Physiol. 2026 Mar 9;17:1730157. doi: 10.3389/fphys.2026.1730157 (PMC13006304; doi:10.3389/fphys.2026.1730157)
Supplement: Supplementary file 1 [file Table1.docx]

**Supplementary Tables**

Table S1. Two-way interactions of hen age, dietary P, and hen strain on concentrations of inositol phosphates and InsP_6_ disappearance in the excreta (P− treatment: n = 99-100 hens and P+ treatment: n = 10 hens, hen age × hen strain: n = 55 hens; n = 219 hens in total).

|  |  |  | Ins(1,2,3,4)P_4_ | Ins(1,2,3,4,6)P_5_ | Ins(1,2,3,4,5)P_5_ | Ins(1,2,4,5,6)P_5_ | InsP_6_ | InsP_6_ disappearance | Ca | P |
| --- | --- | --- | --- | --- | --- | --- | --- | --- | --- | --- |
| Hen age | Dietary P | Hen strain | *µ*mol/g | | | | | % | g/kg DM | |
|  | P−^1^ | LB^3^ | n.d.^5^ | 0.2 | 1.1 | 1.4 | 24.8 | 20.6 | 45.3 | 9.8 |
|  | P− | LSL^4^ | 0.1 | 0.4 | 1.2 | 1.5 | 26.9 | 15.4 | 43.1 | 10.1 |
|  | P+^2^ | LB | n.d. | 0.4 | 1.2 | 2.0 | 26.9 | 17.2 | 48.4 | 13.1 |
|  | P+ | LSL | n.d. | 0.6 | 1.3 | 2.0 | 28.9 | 13.3 | 44.9 | 12.7 |
|  |  | Pooled SEM P− | . | 0.03 | 0.02 | 0.04 | 0.35 | 1.06 | 1.55 | 0.10 |
|  |  | Pooled SEM P+ | . | 0.07 | 0.04 | 0.08 | 0.71 | 2.37 | 3.06 | 0.26 |
|  |  |  |  |  |  |  |  |  |  |  |
| 30 | P− |  | 0.1 | 0.3 | 1.2 | 1.7 | 27.3 | 12.7 | 45.4 | 10.3 |
| 30 | P+ |  | n.d. | 0.5 | 1.4 | 2.4 | 29.2 | 12.9 | 49.1 | 13.5 |
| 42 | P− |  | n.d. | n.d. | 1.0 | 1.2 | 24.3 | 23.3 | 43.1 | 9.6 |
| 42 | P+ |  | n.d. | n.d. | 1.1 | 1.7 | 26.6 | 17.6 | 44.1 | 12.3 |
|  |  | Pooled SEM P− | . | 0.02 | 0.02 | 0.04 | 0.33 | 0.92 | 1.91 | 0.09 |
|  |  | Pooled SEM P+ | . | 0.06 | 0.04 | 0.08 | 0.71 | 2.31 | 3.24 | 0.26 |
|  |  |  |  |  |  |  |  |  |  |  |
| 30 |  | LB | n.d. | 0.3 | 1.2 | 2.0 | 26.9 | 14.3 | 49.4 | 11.5^b^ |
| 30 |  | LSL | 0.1 | 0.5 | 1.4 | 2.1 | 29.6 | 11.3 | 45.1 | 12.3^a^ |
| 42 |  | LB | n.d. | n.d. | 1.0 | 1.4 | 24.7 | 23.5 | 44.4 | 11.4^b^ |
| 42 |  | LSL | n.d. | n.d. | 1.1 | 1.4 | 26.1 | 17.5 | 42.8 | 10.5^c^ |
|  |  | pooled SEM | . | 0.05 | 0.03 | 0.07 | 0.62 | 1.96 | 2.85 | 0.21 |

^a-c^Different superscript lowercase letters within a column indicate significant effects of two-way interactions.

^1^P− = without mineral P supplement.

^2^P+ = with 1 g supplement P/kg.

^3^LB = Lohmann Brown-classic.

^4^LSL = Lohmann LSL-classic.

^5^n.d. = not detectable (< 0.1 *µ*mol/g).

Data are given as LSmeans.

Table S2. Main effects of hen age, dietary P, and hen strain on concentrations of inositol phosphates in the excreta and InsP_6_ disappearance of laying hens (30-wk-old: n = 109 and 42-wk-old: n = 110, P− treatment: n = 199-200 hens and P+ treatment: n = 20 hens, LB hens: n = 109 and LSL hens n = 110; n = 219 hens in total).

|  |  |  | Ins(1,2,3,4)P_4_ | Ins(1,2,3,4,6)P_5_ | Ins(1,2,3,4,5)P_5_ | Ins(1,2,4,5,6)P_5_ | InsP_6_ | InsP_6_ disappearance | Ca | P |
| --- | --- | --- | --- | --- | --- | --- | --- | --- | --- | --- |
| Hen age | Dietary P | Hen strain | *µ*mol/g | | | | | % | g/kg DM | |
| 30 |  |  | 0.1 | 0.4 | 1.3^a^ | 2.0^a^ | 28.2^a^ | 12.8^b^ | 47.3 | 11.9^a^ |
| 42 |  |  | n.d.^5^ | n.d. | 1.1^b^ | 1.4^b^ | 25.4^b^ | 20.5^a^ | 43.6 | 10.9^b^ |
|  |  | Pooled SEM | . | 0.03 | 0.02 | 0.05 | 0.46 | 1.40 | 2.33 | 0.15 |
|  |  |  |  |  |  |  |  |  |  |  |
|  | P−^1^ |  | 0.1 | 0.3^b^ | 1.1^b^ | 1.4^b^ | 25.8^b^ | 18.0 | 44.2 | 9.9^b^ |
|  | P+^2^ |  | n.d. | 0.5^a^ | 1.3^a^ | 2.0^a^ | 27.9^a^ | 15.3 | 46.6 | 12.9^a^ |
|  |  | Pooled SEM P− | . | 0.02 | 0.01 | 0.03 | 0.26 | 0.76 | 1.37 | 0.07 |
|  |  | Pooled SEM P+ | . | 0.06 | 0.03 | 0.06 | 0.54 | 1.75 | 2.41 | 0.19 |
|  |  |  |  |  |  |  |  |  |  |  |
|  |  | LB^3^ | n.d. | 0.3^b^ | 1.1^b^ | 1.7 | 25.8^b^ | 18.9^a^ | 46.9 | 11.4 |
|  |  | LSL^4^ | n.d. | 0.5^a^ | 1.3^a^ | 1.8 | 27.9^a^ | 14.4^b^ | 44.0 | 11.4 |
|  |  | pooled SEM | . | 0.05 | 0.02 | 0.06 | 0.47 | 1.48 | 2.04 | 0.15 |

^a,b^Different superscript lowercase letters within a column indicate significant effects of main effect.

^1^P− = without mineral P supplement.

^2^P+ = with 1 g supplement P/kg.

^3^LB = Lohmann Brown-classic.

^4^LSL = Lohmann LSL-classic.

^5^n.d. = not detectable (< 0.1 *µ*mol/g).

Data are given as LSmeans.

Table S3. Two-way interactions of hen age, dietary P, and hen strain on concentrations of inositol phosphates in the ileum digesta. (P− treatment: n = 99-100 hens and P+ treatment: n = 10 hens, hen age × hen strain: n = 55 hens; n = 219 hens in total)

|  |  |  | MI^1^ | Ins(1,2,3,4,6)P_5_ | Ins(1,2,3,4,5)P_5_ | Ins(1,2,4,5,6)P_5_ | InsP_6_ |
| --- | --- | --- | --- | --- | --- | --- | --- |
| Hen age | Dietary P | Hen strain | *µ*mol/g | | | | |
|  | P−^2^ | LB^4^ | 3.9 | 0.4^a^ | 1.2 | 1.7 | 34.7^a^ |
|  | P− | LSL^5^ | 2.1 | 0.4^a^ | 1.1 | 1.5 | 31.7^b^ |
|  | P+^3^ | LB | 2.7 | 0.3^a^ | 1.0 | 1.9 | 29.3^b^ |
|  | P+ | LSL | 1.4 | n.d.^a,6^ | 1.2 | 1.9 | 34.3^a,b^ |
|  |  | Pooled SEM P− | 0.22 | 0.02 | 0.03 | 0.06 | 0.75 |
|  |  | Pooled SEM P+ | 0.41 | 0.03 | 0.07 | 0.11 | 1.72 |
|  |  |  |  |  |  |  |  |
| 30 | P− |  | 2.8 | 0.3 | 1.1 | 1.6 | 34.1 |
| 30 | P+ |  | 2.1 | n.d. | 1.0 | 1.9 | 30.9 |
| 42 | P− |  | 3.3 | 0.5 | 1.2 | 1.6 | 32.3 |
| 42 | P+ |  | 2.0 | 0.5 | 1.2 | 1.9 | 32.7 |
|  |  | Pooled SEM P− | 0.25 | 0.02 | 0.04 | 0.08 | 1.10 |
|  |  | Pooled SEM P+ | 0.55 | 0.04 | 0.09 | 0.16 | 2.42 |
|  |  |  |  |  |  |  |  |
| 30 |  | LB | 3.3 | 0.3 | 1.1 | 2.0^a^ | 33.8^a^ |
| 30 |  | LSL | 1.5 | n.d. | 1.0 | 1.5^b^ | 31.2^a^ |
| 42 |  | LB | 3.3 | 0.4 | 1.1 | 1.6^a,b^ | 30.2^a^ |
| 42 |  | LSL | 2.0 | 0.5 | 1.3 | 1.9^a^ | 34.7^a^ |
|  |  | pooled SEM | 0.48 | 0.04 | 0.08 | 0.14 | 2.02 |

^a,b^Different superscript lowercase letters within a column indicate significant effects of two-way interactions.

^1^MI = *myo*-inositol.

^2^P− = without mineral P supplement.

^3^P+ = with 1 g supplement P/kg.

^4^LB = Lohmann Brown-classic.

^5^LSL = Lohmann LSL-classic.

^6^n.d. = not detectable (< 0.1 *µ*mol/g).

Data are given as LSmeans.

Table S4. Main effects of hen age, dietary P, and hen strain on concentrations of inositol phosphates in the ileum digesta. (30-wk-old: n = 109 and 42-wk-old: n = 110, P− treatment: n = 199-200 hens and P+ treatment: n = 20 hens, LB hens: n = 109 and LSL hens n = 110; n = 219 hens in total).

|  |  |  | MI^1^ | Ins(1,2,3,4,6)P_5_ | Ins(1,2,3,4,5)P_5_ | Ins(1,2,4,5,6)P_5_ | InsP_6_ |
| --- | --- | --- | --- | --- | --- | --- | --- |
| Hen age | Dietary P | Hen strain | *µ*mol/g | | | | |
| 30 |  |  | 2.4 | n.d.^6^ | 1.1^b^ | 1.7 | 32.5 |
| 42 |  |  | 2.6 | 0.5 | 1.2^a^ | 1.8 | 32.5 |
|  |  | Pooled SEM | 0.35 | 0.02 | 0.06 | 0.11 | 1.54 |
|  |  |  |  |  |  |  |  |
|  | P−^2^ |  | 3.0^a^ | 0.4 | 1.1 | 1.6 | 33.2 |
|  | P+^3^ |  | 2.0^b^ | n.d. | 1.1 | 1.9 | 31.8 |
|  |  | Pooled SEM P− | 0.16 | 0.01 | 0.02 | 0.05 | 0.64 |
|  |  | Pooled SEM P+ | 0.31 | 0.00 | 0.05 | 0.09 | 1.32 |
|  |  |  |  |  |  |  |  |
|  |  | LB^4^ | 3.3^a^ | 0.3 | 1.1 | 1.8 | 32.0 |
|  |  | LSL^5^ | 1.8^b^ | n.d. | 1.2 | 1.7 | 33.0 |
|  |  | pooled SEM | 0.28 | 0.01 | 0.04 | 0.07 | 1.08 |

^a,b^Different superscript lowercase letters within a column indicate significant effects of main effect.

^1^MI = *myo*-inositol.

^2^P− = without mineral P supplement.

^3^P+ = with 1 g supplement P/kg.

^4^LB = Lohmann Brown-classic.

^5^LSL = Lohmann LSL-classic.

^6^n.d. = not detectable (< 0.1 *µ*mol/g).

Data are given as LSmeans.

Table S5. Two-way interactions of hen age, dietary P, and hen strain on concentrations of inositol phosphates in the incubation residue and InsP_6_ disappearance in the three-step *in vitro* assay with freeze-dried mucosa of laying hens (P− treatment: n = 99-100 hens and P+ treatment: n = 10 hens, hen age × hen strain: n = 55 hens; n = 219 hens in total).

|  |  |  | InsP_3x_^1^ | Ins(1,2,3,4)P_4_ | Ins(1,2,3,4,6)P_5_ | Ins(1,2,3,4,5)P_5_ | Ins(1,2,4,5,6)P_5_ | InsP_6_ | InsP_6_ disappearance | InsP_<3_^2^ |
| --- | --- | --- | --- | --- | --- | --- | --- | --- | --- | --- |
| Hen age | Dietary P | Hen strain | *µ*mol/g | | | | | | % | *µ*mol/g |
|  | P−^3^ | LB^5^ | 2.4^b^ | 1.4^b^ | <LOQ^7^ | 0.4 | <LOQ | 5.0 | 53.9 | 2.4 |
|  | P− | LSL^6^ | 2.6^a^ | 1.5^b^ | <LOQ | 0.4 | <LOQ | 4.9 | 54.8 | 2.4 |
|  | P+^4^ | LB | 1.5^d^ | 1.3^b^ | <LOQ | 0.7 | 0.2 | 6.4 | 41.4 | 1.6 |
|  | P+ | LSL | 2.1^c^ | 1.7^a^ | <LOQ | 0.6 | <LOQ | 5.9 | 45.7 | 1.4 |
|  |  | Pooled SEM P− | 0.07 | 0.04 | . | 0.03 | . | 0.12 | 1.07 | 0.11 |
|  |  | Pooled SEM P+ | 0.13 | 0.09 | . | 0.05 | . | 0.23 | 2.08 | 0.25 |
|  |  |  |  |  |  |  |  |  |  |  |
| 30 | P− |  | 2.5 | 1.4 | <LOQ | 0.4 | n.d.^8^ | 5.0^c^ | 54.0^a^ | 2.5 |
| 30 | P+ |  | 1.6 | 1.4 | <LOQ | 0.7 | 0.2 | 6.6^a^ | 39.4^c^ | 1.3 |
| 42 | P− |  | 2.6 | 1.5 | <LOQ | 0.5 | <LOQ | 4.9^c^ | 54.6^a^ | 2.3 |
| 42 | P+ |  | 1.9 | 1.6 | <LOQ | 0.7 | <LOQ | 5.7^b^ | 47.8^b^ | 1.7 |
|  |  | Pooled SEM P− | 0.07 | 0.04 | . | 0.02 | . | 0.12 | 1.09 | 0.10 |
|  |  | Pooled SEM P+ | 0.13 | 0.09 | . | 0.05 | . | 0.23 | 2.09 | 0.25 |
|  |  |  |  |  |  |  |  |  |  |  |
| 30 |  | LB | 1.8 | 1.2 | <LOQ | 0.6 | 0.2 | 6.0 | 44.9 | 1.9 |
| 30 |  | LSL | 2.3 | 1.5 | <LOQ | 0.5 | n.d. | 5.6 | 48.5 | 1.9 |
| 42 |  | LB | 2.1 | 1.5 | <LOQ | 0.6 | <LOQ | 5.4 | 50.4 | 2.1 |
| 42 |  | LSL | 2.4 | 1.6 | <LOQ | 0.5 | <LOQ | 5.2 | 52.0 | 1.9 |
|  |  | Pooled SEM | 0.12 | 0.07 | . | 0.05 | . | 0.20 | 1.84 | 0.21 |

^a-d^Different superscript lowercase letters within a column indicate significant effects of two-way interactions.

^1^InsP_3x_ = Ins(1,2,6)P_3_, Ins(1,4,5)P_3_, and Ins(2,4,5)P_3_ could not be differentiated due to co-elution and are thus referred to as InsP_3x_.

^2^InsP_<3_ = fraction of InsP isomers lower than InsP_3x_ after *in vitro* incubation.

^3^P− = without mineral P supplement.

^4^P+ = with 1 g supplement P/kg.

^5^LB = Lohmann Brown-classic.

^6^LSL = Lohmann LSL-classic.

^7^<LOQ = below limit of quantification (for Ins(1,2,5,6)P_4_ and Ins(1,2,3,4,6)P_5_ 0.3 *µ*mol/g and for Ins(1,2,4,5,6)P_5_ 0.2 *µ*mol/g).

^8^n.d. = not detectable (< 0.1 *µ*mol/g).

Data are given as LSmeans.

|  |  |  | InsP_3x_^1^ | Ins(1,2,3,4)P_4_ | Ins(1,2,3,4,6)P_5_ | Ins(1,2,3,4,5)P_5_ | Ins(1,2,4,5,6)P_5_ | InsP_6_ | InsP_6_ disappearance | InsP_<3_^2^ |
| --- | --- | --- | --- | --- | --- | --- | --- | --- | --- | --- |
| Hen age | Dietary P | Hen strain | *µ*mol/g | | | | | | % | *µ*mol/g |
| 30 |  |  | 2.0 | 1.4 | <LOQ^7^ | 0.6 | 0.2 | 5.8 | 46.7 | 1.9 |
| 42 |  |  | 2.3 | 1.6 | <LOQ | 0.6 | <LOQ | 5.3 | 51.2 | 2.0 |
|  |  | Pooled SEM | 0.09 | 0.06 | . | 0.03 | . | 0.15 | 1.41 | 0.15 |
|  |  |  |  |  |  |  |  |  |  |  |
|  | P−^3^ |  | 2.5 | 1.4 | <LOQ | 0.4^b^ | <LOQ | 5.0 | 54.3 | 2.4^a^ |
|  | P+^4^ |  | 1.8 | 1.5 | <LOQ | 0.7^a^ | 0.2 | 6.1 | 43.6 | 1.5^b^ |
|  |  | Pooled SEM P− | 0.06 | 0.03 | . | 0.02 | . | 0.09 | 0.85 | 0.08 |
|  |  | Pooled SEM P+ | 0.10 | 0.07 | . | 0.04 | . | 0.17 | 1.59 | 0.19 |
|  |  |  |  |  |  |  |  |  |  |  |
|  |  | LB^5^ | 1.9 | 1.4 | <LOQ | 0.6 | 0.2 | 5.7 | 47.6 | 2.0 |
|  |  | LSL^6^ | 2.3 | 1.6 | <LOQ | 0.5 | <LOQ | 5.4 | 50.3 | 1.9 |
|  |  | Pooled SEM | 0.09 | 0.05 | . | 0.04 | . | 0.15 | 1.40 | 0.16 |

Table S6. Main effects of hen age, dietary P, and hen strain on concentrations of inositol phosphates in the incubation residue and InsP_6_ disappearance in the three-step *in vitro* assay with freeze-dried mucosa of laying hens (30-wk-old: n = 109 and 42-wk-old: n = 110, P− treatment: n = 199-200 hens and P+ treatment: n = 20 hens, LB hens: n = 109 and LSL hens n = 110; n = 219 hens in total).

^a,b^Different superscript lowercase letters within a column indicate significant effects of main effect.

^1^InsP_3x_ = Ins(1,2,6)P_3_, Ins(1,4,5)P_3_, and Ins(2,4,5)P_3_ could not be differentiated due to co-elution and are thus referred to as InsP_3x_.

^2^InsP_<3_ = fraction of InsP isomers lower than InsP_3x_ after *in vitro* incubation.

^3^P− = without mineral P supplement.

^4^P+ = with 1 g supplement P/kg.

^5^LB = Lohmann Brown-classic.

^6^LSL = Lohmann LSL-classic.

^7^<LOQ = below limit of quantification (for Ins(1,2,5,6)P_4_ and Ins(1,2,3,4,6)P_5_ 0.3 *µ*mol/g and for Ins(1,2,4,5,6)P_5_ 0.2 *µ*mol/g).

Data are given as LSmeans.
